# False Impressions

**Published:** 1889-01-26

**Authors:** Caroline Fothergill

**Affiliations:** Author of "An Enthusiast," "A Voice in the Wilderness," etc.


*(To be continued.)*

### FALSE IMPRESSIONS.

By Caroline Fothergill, Author of "An Enthusiast," "A Voice in the Wilderness," etc.

*(Continued from p. 257.)*

### Chapter XVI.—Suspicions.

A day or two after the evening at the Museum, Beatrice and Laura called on Mrs. Lester. This exchange of facilities was very distasteful to Beatrice, and could she have avoided it she would gladly have done so. But even she, with her natural fondness, which had been fostered by outside circumstances, of making things bend to her will, did not see how she could neglect to pay this call, and she went. She came off better than she had expected. George was out, nor did he come in during the twenty minutes of her stay. Only Mrs. Lester and Edith were present. If Edith could have had her way she would have sat in the background, taking no share in the conversation, but observing these two women who interested her so much; for, added to her interest in Beatrice, was her natural shyness, and her dread of Mrs. Ledward. But she was not allowed to follow her own wishes; her aunt made her a peremptory sign which she dared not disobey, and Laura, whose bright eyes had been roving all over the room from the moment she had entered it, drew near to Edith and made her talk in spite of herself.

She was relieved to find that Miss Stanford scarcely noticed her. She was making the call under compulsion, and was bent upon getting it over as quickly as possible. She did just offer her hand to Edith when she heard her name, but that was all. She at once directed her attention to Mrs. Lester, who was by no means in an amiable mood, and, as her brother was not by, she quickly made up her mind to give Miss Stanford a hint as to the utter uselessness of seeking an intimacy with the household in Queen Street. Beatrice had made some remark about going from home, and had asked Mrs. Lester if she were going into the country at all.

Mrs. Lester replied that she could not tell. Her plans were always entirely dependent upon those of her brother. She knew she was indispensable to his comfort and happiness, but she had never abused her position by exacting a liberty which might inconvenience him.

"Although I am a widow," she went on, "and my husband was the best husband woman ever had, I often say that if I were a man I should remain a bachelor. I can so well understand all they have to give up when they marry, and the cares and responsibilities with which they burden themselves."

Mrs. Lester, who, finding conversation with Edith rather uphill work, had abandoned the attempt in despair, overheard the last words, and had to raise her handkerchief to her face to conceal the flash of mirth which passed over it.

"I am afraid yours was an unfortunate experience," she said, as soon as Mrs. Lester had finished speaking. "I am a widow too; but the recollection of my married life leads me to advocate marriage for all whose character does not absolutely disqualify them from living happily with another person."

Mrs. Lester frowned, but was not silenced.

"Oh, yes," she said; "some people are very easy-going. My brother is not. I can imagine anyone who did not understand him would irritate him beyond measure, and make both him and herself positively miserable. It would be a very serious thing for any woman to undertake to make him happy; nine out of ten would fail completely. No; I must say he shows his wisdom in remaining with his old sister, who understands his whims and moods."

To these observations Beatrice made no reply. She would have risen to go at once, but Laura, who was enjoying it, threw her an imploring glance, to which she yielded, and, leaving the two widows to sharpen their wits on one another, she turned to Edith, and began to talk to her. She spoke in the imperious tone which she almost unconsciously employed when she was annoyed, and which had the effect of frightening Edith almost out of her senses, and reducing her conversational powers to "Yes" and "No." She began to think Miss Stanford a most formidable person; but as long as she left her (Edith) alone there was comfort in that, for she was sure such a woman would never attract Mr. Murray, who had shown himself so susceptible to Edith's milder charms. To be sure, Miss Stanford was very beautiful, and her dress was the perfection of costly and artistic simplicity; but by one of those rare flashes of insight which sometimes come so oddly to people of little experience, she at once grasped the spirited, self-reliant side of Beatrice's nature, and decided in her own mind that those qualities would rather repel than attract Mr. Murray, which we know was at least partially true. Still she was glad when Miss Stanford rose decidedly, and took leave of her. Mrs. Ledward departed unwillingly; she was enjoying herself thoroughly, and as soon as she and Beatrice were again in the carriage she threw herself back, and gave full vent to her long pent-up amusement.

"A truly unique household," she said at last. "It is long since I so enjoyed an afternoon call, even though we got no tea. A thousand pities Mr. Murray did not come in; he could only have added to the picturesqueness of the situation. I hoped every footstep I heard was his."

"I am sorry you were disappointed. For my part, I thought it just as well he was away. His sister is a terribly vulgar woman."

"Yes," said Laura; "you did not seem to get on with Mrs. Lester at all. How was it? I thought her so amusing. You seemed equally unsuccessful with Edith."

Beatrice made no answer, and Laura went on:

"Well, I suppose, now the acquaintance is fairly established, you will have to ask them to dinner."

"I shall not ask them to dinner."

"Well, no; after all, they will probably ask us first."

"If they do, we need not go. I do not think I shall ever enter the house again."

Laura said nothing, but indulged in a grimace and an expressive shrug of the shoulders.

George said very little when he heard at dinner who had called in his absence. His sister described the visit with much complacency. She had recovered her temper, and flattered herself that she had settled matters for a time, at any rate. George only half heard all she said. He had made up his mind that the acquaintance should not end here. He wanted to know Miss Stanford, and he would know her. She affected him differently from any other woman he had ever known, and he was not going to be deprived of the pleasure of intercourse with her. Almost insensibly his opinion respecting her had grown to be the very opposite of what it had been at the beginning. One little incident after another had happened; one after another he had had occasion to observe certain characteristics which she possessed; and he was influenced, too, by a nameless something which he could neither describe nor analyse, but which really affected him more strongly than any of the more tangible reasons which he had. He began to consider how he might best see her again, how strengthen the bond which already existed between them. He thought of a great many plans, but rejected them all. After all, they lived in the nineteenth century, and were bound by social conventions. He must proceed in the usual way; nothing would be gained by deviating from the trodden path. But he would act at once in the matter; of that he was determined.

"Frances," he said, as Edith poured out tea that same evening, "it is a long time since we had a dinner party. I think it is time we had another."

"It is not the time of year for dinner parties, in Mayfield, at any rate," replied Mrs. Lester.

"Yes it is, if we choose to give one. I should like to have one. Let us send out the invitations to-morrow."

"You are very changeable," said his sister, though as yet without suspicion as to the reason of the change. "You have often said what a bore it was having people to dinner, and have put obstacles in my way when I proposed it."

"I have changed my mind. I should like a party now. Let us choose our guests. Edith, get a pencil and paper, and write down the names as we decide upon them."

Edith was always happy to do anything for George, and of late he had often asked her to do things for him, and she, pleased to show what a help-meet could be, had always joyfully accepted his tasks. Now she got pencil and paper without delay, and the somewhat difficult task of selecting their guests from their very large circle of acquaintance began.

At first all went smoothly. Half-a-dozen, on whose merits Mrs. Lester and her brother were perfectly agreed, were chosen, and then George said:

"Miss Stanford and Mrs. Ledward."

"No!" exclaimed Mrs. Lester, energetically, almost bounding from her seat as she spoke.

It was more an ejaculation than a contradiction, and was followed by silence. Edith looked up almost frightened, and George stood looking at his sister with raised eyebrows.

"I beg your pardon," he said, with perfect politeness, but at the same time with surprise.

By this time Mrs. Lester had recovered her presence of mind and also her equanimity, and when she spoke again it was in a quieter tone.

"I think it will be a mistake to invite them," she said. "All the other women are married, and bring their own men with them; it is far less trouble to invite only married people."

"Yes, but it is dull and monotonous," said George. "We have gone on in a groove too long; we want a change. Edith and I, being unmarried ourselves, prefer at least some of our guests to be unmarried too. Don't we, Edith?"

Edith was too frightened at her aunt's evident anger to say anything; besides, she only seriously cared about one unmarried man. So she only smiled and looked down at the list of names which already stood upon her paper.

"I think," went on Mrs. Lester, desperately, giving the first reasons which came into head, "that you will be placing Miss Stanford in an awkward position by inviting her. Ten to one she will not know anyone here."

"My dear Frances," said George, smiling, "Miss Stanford is not a girl of seventeen, whom we are inviting to her first dinner party."

"We shall have to find two odd men to take them in."

"There will be no difficulty about that. Where am I, for one; and in any case we should have had to get someone for Edith."

"Edith has never dined with us."

"She must begin, then. She shall begin this very time, and if we are ashamed of having a dinner party in July, we can make use of Edith's first appearance to give colour to our proceeding. That will do excellently."

Mrs. Lester was nearly frantic at this realization of her fears. She foresaw what was going to happen, and was ready to use all means to prevent it. Unfortunately her manner on such occasions was not happy, and she more frequently alienated than conciliated people. She did not this evening depart from her custom.

"As for taking in Miss Stanford," she said, "it is absurd. You cannot possibly do it with both Mrs. Hunter and Mrs. Williamson here"

"I shall take in Miss Stanford, whoever is here."

"You cannot, George," her voice vibrating with restrained anger. "They are both old friends of mine, and for a whim you risk causing an estrangement. You will place me in a most awkward position."

"Nonsense," he said, lightly. "If you wish to be strictly accurate, Miss Stanford would anywhere take precedence of either of the ladies you name. Do not say any more, I beg; my mind is made up."

She knew it. She knew that defeat was her portion, and she dared say no more. But her anger and apprehension were too great to allow her to keep silence, and after a pause she burst out again.

"That odious little Mrs. Ledward, too; I hate her."

"I like her," said George. "She is very good company. I shall be glad to see her again."

Mrs. Lester said no more. She was sore and smarting with her defeat, and she knew she had made herself thoroughly disagreeable. Of course she had gained nothing by opposing her brother. She was furious with both herself and him—and Mrs. Lester's furies never failed to have results.

